# The Rôle of Dilute Acids in Infection

**Published:** 1921-12

**Authors:** I. Walker Hall, A. D. Fraser


### THE RÔLE OF DILUTE ACIDS IN INFECTION.

BY

I. WALKER HALL AND A. D. FRASER.

---

THE tendency of modern thought is to probe into the infinitely little. In physics, chemistry and bio-chemistry the older ideas are being re-clothed to fit newer conceptions. Medicine is exhorted to focus attention on the beginnings of disease.

This changing frame of mind makes exhausting demands upon the methods available for such observations. Progress has to wait for the provision of efficient technique. Hurry spells wasted effort ; for he who examines in detail the technics of the day or visits the "midden heaps" of abandoned processes and discarded data soon understands the utility of making haste slowly. In the laboratory area of scientific medicine, at all events, there is hardly a field which has not first to be harrowed with the testing and improvement of methods. Bacteriology, bio-chemistry, micro-chemistry alike stand hesitating, not daring to make undue applications of the results of current work.

One of the basal factors of experiment and observation is the estimation of neutrality. A piece of litmus paper no longer suffices. Even the titration of total acidity, or alkalinity, fails to yield adequate information. A knowledge of the intensity of the reaction, or ion concentration is also required.

When sodium chloride is added to water to make normal saline solution, the sodium and the chloride separate into ions, or sub-molecular groups charged with electrical functions. If HCl is substituted, the acid solution contains hydrogen ions and chloride ions. The H ion loses a negative charge and becomes positively attractive: while the Cl ion gains a negative charge. These H ions circulating in animal tissues act as accelerators, or catalysers of the metabolic processes of tissue cells. Each cell of the body requires an environment of H ions ranging from 0,000,000,1 to 0,000,000,01 grams H per litre of tissue fluids for its normal work. If these limits are exceeded, abnormal changes result. The cell colloids, such as globulins, are not properly hydrated and cloudy swelling follows. These ions are appropriately designated as the workmen of the cells and by control of enzyme action influence the metabolic exchanges. It is obvious, therefore, that accurate determination of the ion capacity, or concentration, is a necessity for advances in the knowledge of cellular processes.

Taking absolutely pure water as exact neutrality, its position in the scale of reactions is represented for convenience by the index, pH 7. Blood is a trifle alkaline, pH 7·4. The muscles yield a pH of 6·9, urine 5·6, gastric juice 0·9–1·6, all being more acid than blood.

One of the chief functions of the blood is to maintain a general degree of tissue reaction, in spite of the local differences and of its continuous receipt of acids and bases from the food, and carbonic, phosphoric, sulphuric and lactic acids produced during ordinary metabolism. It achieves this by reason of its content of proteins, phosphates and bicarbonates which neutralise or " buffer " the acid materials.

In disease there are also local acidities which increase, this work of the blood. In acute abscesses the pH is 5·9, in chronic abscesses 6·5, in ascitic fluid 7·6. During the stage of resolution of lobar pneumonia the absorption of the exudate depends upon changes in acidity, from pH 7 to pH 6·5, which determine the extent of necessary enzymatic actions.[1]

While we were investigating the technics upon which these figures were based we found that they had been applied to a few acids only. It was necessary, therefore, to determine how far they could be utilised for all types of mineral and organic acids, and especially for those that are built up within the body tissues during normal and abnormal processes. As this demanded the co-operation of the physical chemist, we turned to Professor McBain for help. He kindly assisted us with discussion and criticism and allowed Miss Kieser, one of his honours students, to check independently our own findings. These physical measurements showed that our methods were available and accurate for a wide range of acid substances.

On this basis we extended our observations upon pH influences obtained from acids of high dissociating capacities and also from those that dissociated but slightly, leaving in solution a large proportion of the unaltered acids, such as the monobasic, di-basic, hydroxy and polyvalent fatty acids.

We soon found that when bacteria are subjected to minute alterations of acidity obtained by the use of different types of acids, their growth may be accelerated or retarded.[2] For instance, 1/200 n/nitric acid or 1/200 n/lactic stimulates the growth of streptococci, while 1/100 n/citric acid retards it. And so on throughout the whole range of pyogenic and other organisms there are to be found H ions with the other ions, or anions—in this case, nitrates and lactates—which catalyse the ordinary growth of the organisms. With diphtheria bacilli, phosphoric, lactic and nitric derived ions act as stimulants, whereas the saturated monobasic fatty acids, such as formic, butyric and propionic, retard development.

When it is remembered, that the pH of the duodenal contents controls the prepyloric sphincter and the rate of outflow from the stomach, and when we point out that sputum contains areas varying in their pH content, and that local pH alterations are necessary for the effective performance of physiological functions as well as for adequate response to infections, it is evident that a careful survey of the associated factors is called for. There are the questions of the influence of minute pH ranges in the tissues as they affect the acceleration or retarding during bacterial invasion: the possibility of inhibiting the growth of organisms in purulent conditions by altering the pH of the exudate: the conditioning of local areas so that diphtheria bacilli cannot attain their necessary alkaline surroundings for the production of toxin: and a damaging pH of tissues so that the diagnosis of experimental inoculations may be hastened.

Much claims that by injecting lactic acid together with saprophytic bacteria into animals he can turn a harmless germ into a pathogen.[3] Our experiments so far have not confirmed this, but we have been able to raise the virulence of pneumococci by similar procedures.

Although these investigations are in their preliminary stages and are not communicated for purposes of criticism and information, there has emerged a very important application

13

in the improvements of the technique of blood cultures. By the use of altering the pH of the blood media, we have obtained an earlier and higher percentage of positive results and previsioned a means of concomitant estimation of the exact bacteriolytic resistance manifested by the patient.

As we look at infectious processes in the light gained by a study of these various forms of acidification, we begin to understand how there is also to be considered the penetrability and permeability of the individual acids. The knowledge of the varying permeability of red and white corpuscles in health and disease points the way to a closer acquaintance with similar functions in cells lining serous cavities and tissue spaces. The recent work of Crozier, Haas and others indicates the high penetrability possessed by lactic and mineral acids.[4, 5] This throws another ray of illumination upon the primary or secondary features which are met with in oral and intestinal sepsis. If the presence of very minute quantities of dilute acids affects bacterial growth on the one hand and influences penetrability and permeability on the other one, we may look at infection from another angle and re-enter obscure fields of enquiry with an additional weapon for attack.

### REFERENCES.

[1] Lord, F. T., *J. Exp. Med.*, 1919, xxx. 379.

[2] I. Walker Hall and A. D. Fraser, *Brit. J. Exp. Path.*, 1921, v. 242, and *J. Path.*, 1922, xxv.

[3] Much, H., *Deutsche med. Wchnschr.*, 1921, xlvii. 621.

[4] Crozier, W. J., *J. Biol. Chem.*, 1916, xxiv. 255.

[5] Haas, A., *J. Biol. Chem.*, 1916, xxvii. 225.

